# Functional Capacity and Exercise Tolerance in Residential Care Home Residents with Treated Arterial Hypertension and Coronary Heart Disease

**DOI:** 10.3390/jcm15155916

**Published:** 2026-07-29

**Authors:** Małgorzata Fortuna, Antonina Kaczorowska, Jacek Szczurowski, Zofia Ignasiak

**Affiliations:** 1 Faculty of Health and Physical Culture Sciences, Witelon Collegium State University in Legnica, 59-220 Legnica, Poland; 2 Institute of Health Sciences, University of Opole, 45-040 Opole, Poland; antonina.kaczorowska@uni.opole.pl; 3 Department of Anthropology, Wroclaw University of Environmental and Life Sciences, 50-139 Wrocław, Poland; jacek.szczurowski@upwr.edu.pl; 4 Faculty of Medicine, Department of Preclinical Sciences, Pharmacology and Medical Diagnostics, Wrocław University of Science and Technology, 50-370 Wrocław, Poland; zofia.ignasiak@pwr.edu.pl

**Keywords:** functional capacity, exercise tolerance, older adults, residential care, SPPB, 6MWT

## Abstract

**Background**: Older adults commonly experience a progressive decline in functional capacity and exercise tolerance, particularly among residents of residential care homes. Advanced age is associated with a higher prevalence of coronary heart disease (CHD) and arterial hypertension, both of which further reduce physical activity levels. Evidence indicates that regular, structured physical activity can improve physical functioning and cardiovascular parameters. This study aimed to assess functional capacity and exercise tolerance in residential care home residents with treated arterial hypertension and CHD compared with individuals without cardiovascular disease. **Methods**: The study included 74 men aged 65–80 years residing in residential care homes. Participants were divided into two groups: Group I consisted of 44 men without diagnosed cardiovascular disease, and Group II included 30 men with diagnosed CHD and arterial hypertension. Functional capacity was assessed using the Short Physical Performance Battery (SPPB), and exercise tolerance was evaluated using the 6-Minute Walk Test (6MWT). **Results**: The mean SPPB score was 8.7 ± 2.1 points in Group I and 5.2 ± 2.5 points in Group II (*p* < 0.001). In the 6 MWT, participants in Group I covered a mean distance of 312.7 ± 89.4 m, whereas those in Group II covered 168.4 ± 84.4 m (*p* < 0.001). Multivariable regression analysis confirmed that group allocation remained an independent predictor of both SPPB and 6MWT outcomes after adjusting for age and somatic parameters (*p* < 0.001). **Conclusions**: Residential care home residents with CHD and arterial hypertension demonstrate significantly lower functional capacity and exercise tolerance compared with individuals without cardiovascular disease. Both groups achieved results markedly below national reference values for the Polish older adult population. These findings highlight the need for targeted physical activity programs in residential care homes, with particular emphasis on individuals with cardiovascular disease.

## 1. Introduction

The ageing process is associated with a progressive decline in functional capacity and aerobic endurance [1]. Functional capacity is a key prognostic indicator of health status and a fundamental determinant of independence and quality of life in older adults. Its deterioration leads to reduced autonomy, which negatively affects psychological and social functioning [2]. Aerobic capacity, a major component of cardiorespiratory fitness, is most commonly assessed using VO_2max_, metabolic equivalents (METs) achieved during exercise testing, or indirectly through the 6-Minute Walk Test (6MWT) [3]. In adults, VO_2max_ decreases by approximately 8–10% per decade, with the rate of decline accelerating with advancing age. By around 80 years of age, VO_2max_ typically reaches only about 50% of the level observed in early adulthood [4]. Cardiovascular diseases, including arterial hypertension and coronary heart disease (CHD), further exacerbate these unfavorable changes [2].

Ageing is also accompanied by sarcopenia, characterized by a reduction in muscle mass and the number of muscle fibers, leading to decreased muscle strength and an increased relative cost of physical effort. As a result, many older adults withdraw from regular physical activity, which contributes to further deterioration in functional capacity and a faster onset of fatigue [5]. Meanwhile, functional capacity and exercise tolerance are essential for maintaining health and independence in later life [3]. Reduced physical activity leads to diminished physical performance and increased susceptibility to fatigue, whereas regular activity improves physical functioning, enhances self-efficacy, reduces anxiety and depressive symptoms, and lowers stress levels all of which are particularly important in the secondary prevention of cardiovascular disease. Cardiovascular diseases remain the leading cause of morbidity and mortality among older adults and their prevalence increases markedly after the age of 65. In Poland, more than 70% of men over 70 years live with at least one chronic cardiovascular condition, which substantially affects mobility, independence and quality of life. Functional capacity is therefore a critical indicator of health status and a major determinant of successful ageing [6].

Although ageing is one of the main risk factors for cardiovascular diseases, this relationship is bidirectional: low levels of physical activity may accelerate biological ageing and intensify pathological changes [7]. Physical activity and the reduction in sedentary behavior are modifiable factors that, according to WHO recommendations, should be promoted to support healthy ageing [8]. Appropriately designed physical activity programs improve functional capacity, enhance exercise tolerance, reduce the risk of chronic diseases including cardiovascular conditions and support their management [9].

The rate of decline in physical fitness and aerobic capacity varies and depends, among other factors, on physical activity levels and environmental conditions. Among independently living older adults, activity levels often reflect lifestyle and health awareness. In contrast, in residential care homes, physical activity largely depends on the availability of activation programs and the engagement of staff in delivering educational and exercise-based interventions [10]. Identifying residents with reduced functional capacity and exercise tolerance, particularly those with CHD and arterial hypertension, should be an integral component of health-oriented preventive strategies. Despite the clinical relevance of functional assessments, institutionalized older adults remain understudies. Most available research focuses on community-dwelling seniors, whereas residents of long-term care facilities often present lower physical activity levels, greater multimorbidity and limited access to structured exercise programs. Consequently, little is known about the functional capacity and exercise tolerance of older men living in residential care homes, particularly in Central and Eastern Europe. Identifying individuals with reduced functional capacity and exercise tolerance is essential for planning preventive strategies and tailoring physical activity programs in residential care settings. This is particularly important for residents with cardiovascular disease, who may experience accelerated functional decline and higher risk of disability.

The aim of the present study was to assess and compare functional capacity and exercise tolerance in residential care home residents with treated arterial hypertension and CHD versus individuals without cardiovascular disease. Additionally, exercise tolerance was analyzed in relation to Polish population-based normative values.

## 2. Materials and Methods

### 2.1. Research Design and Participants

**Figure 1:**
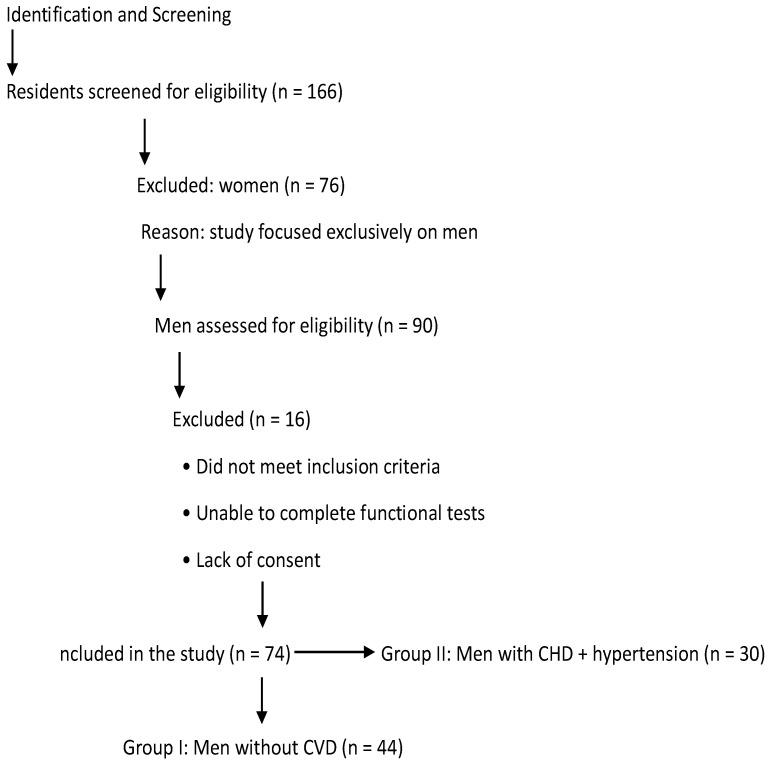
Participant flow diagram.

The study was conducted between 2023 and 2025 in 13 residential care homes located in the southwestern region of Poland. A total of 166 residents (90 men and 76 women) were screened for eligibility. Women were excluded because the study focused exclusively on older men. Among the 90 screened men, 74 met all inclusion criteria and were enrolled in the study. Sixteen men were excluded due to not meeting the health-related inclusion criteria, inability to complete functional tests or lack of consent. The recruitment process and participant flow were described in detail to ensure transparency of the study design (Figure 1).

The study included 74 men aged 65–80 years. The age range of 65–80 years was selected to ensure relative homogeneity in functional and health status within the study population. Individuals over 80 years of age typically present greater variability in health conditions, higher multimorbidity and an increased risk of dependency, which could reduce group comparability and influence function test outcomes. This age range also reflects the typical demographic profile of residential care home residents in Poland and aligns with previous normative studies on functional fitness in older adults. Participants were divided into two groups: Group I: 44 men without diagnosed cardiovascular disease Group II: 30 men with diagnosed and treated coronary heart disease (CHD) and arterial hypertension.

Functional capacity was assessed using the Short Physical Performance Battery (SPPB), and exercise tolerance was evaluated using the 6-Minute Walk Test (6MWT).

Inclusion criteria:

- Men aged 65–80 years;
- Residents of residential care homes in Poland;
- Preserved independent mobility and ability to perform activities of daily living;
- No medical contraindications to functional fitness testing;
- No difficulties in verbal communication;
- No physical or cognitive impairments preventing participation;
- Absence of obesity.

Exclusion criteria:

Exclusion criteria included cancer, acute injuries or infections, recent myocardial infarction, other medical contraindications, and refusal to participate. All participants provided written informed consent prior to enrolment. The study protocol was approved by the Senate Committee on Research Ethics of the University of Opole (approval no. KB/202/FI/2019) and conducted in accordance with the principles of the Declaration of Helsinki.

### 2.2. Methods

Each assessment lasted approximately 30 min and was conducted within a single day. All measurements, including the 6-Minute Walk Test (6MWT) and the Short Physical Performance Battery (SPPB), were performed on-site in the residential care homes. The 6MWT was administered as the final test [11].

### 2.3. Functional Fitness Assessment Procedures

Functional fitness was evaluated using the Short Physical Performance Battery (SPPB), and exercise tolerance was assessed using the 6-Minute Walk Test (6MWT). The SPPB provides information on physical capabilities that reflect the ability to perform basic activities of daily living.

The SPPB assessed functional performance in three domains:

- Lower-limb strength;
- Static balance;
- Gait speed.

### 2.4. Lower-Limb Strength and Endurance

Participants were instructed to rise from a chair with their arms crossed over the chest. If they were able to complete one repetition, they were asked to perform five additional repetitions as quickly as possible. The completion time was used for scoring.

### 2.5. Static Balance Assessment

Participants attempted to maintain stability for 10 s in each of the following positions:

- Feet side-by-side;
- Semi-tandem (heel of one foot touching the big toe of the other);
- Full tandem (one foot placed directly in front of the other, heel-to-toe). Progression to the next position required maintaining the previous stance for 10 s.

### 2.6. Gait Speed Assessment

Participants walked a straight 4 m distance at their fastest safe pace. The recorded time was used for scoring.

SPPB results were interpreted using a standardized scoring system. Each domain was scored from 0 to 4 points (0 = unable to perform, 4 = highest performance). The total score ranged from 0 to 12 points, reflecting overall physical function [12].

### 2.7. Assessment of Exercise Tolerance 6-Minute Walk Test

The 6-Minute Walk Test (6MWT) was used as an indirect measure of aerobic capacity [13]. Participants were instructed to walk as far as possible within six minutes. The distance covered was measured in meters. The test was conducted on a flat, non-slippery surface, along a rectangular course measuring 20 × 5 m.

### 2.8. Somatic Measurements

Body height and weight were measured using a SECA 764 device. Body mass index (BMI) was calculated based on these measurements. The following categories were applied (Table 1):

### 2.9. Statistical Analysis

Statistical analyses were performed using Statistica v.14. After calculating descriptive statistics, the normality of variable distributions was assessed using the Shapiro–Wilk test. Before applying Student’s *t*-test, the assumption of homogeneity of variance was verified using Levene’s test.

Given the distribution characteristics (Table 2), differences in mean values for body weight, height, BMI, 6MWT distance, and SPPB scores were analyzed using the independent samples Student’s *t* test. For the variable age, which demonstrated a non-normal distribution, differences between groups were examined using the Mann–Whitney U test, the non-parametric equivalent of the *t* test.

Variables with normal distribution included:

- Body weight;
- Body height;
- BMI;
- 6MWT distance;
- SPPB score.

Group differences in age were assessed using the Mann–Whitney U test.

A comparison was also performed between the results of the present study and the normative values reported by Ignasiak et al. (2020) [15]. Only age categories corresponding to the study population (men aged 65–80 years) were included to ensure appropriate age- and sex-matching. Distances achieved in the 6MWT were compared with Polish population-based reference values.

In the first stage, weighted means were calculated based on the data provided by Ignasiak et al. (2020) [15]. Age group data were simplified for comparative purposes. Calculations were based on mean values and standard deviations and sample sizes reported in the national normative dataset by Ignasiak et al. (2020) [15].

The weighted mean was calculated using the following formula:

$${\overset{ˉ}{x}}_{\mathrm{weighted}}=\frac{x_{1}w_{1}+x_{2}w_{2}+\cdots +x_{n}w_{n}}{w_{1}+w_{2}+\cdots +w_{n}}$$

where *x*—variable value and *w*—weight (number of individuals in a given age category).

Subsequently, the significance of differences between mean 6MWT distances (present study vs. Ignasiak et al., 2020 [15]) was assessed using a mean difference test (equivalent to the independent samples *t* test).

To determine whether group allocation (presence vs. absence of cardiovascular disease) remained an independent predictor of functional performance, multivariable linear regression analysis was conducted. A significance level of *p* < 0.05 was adopted; 95% confidence intervals for all regression coefficients were calculated using the formula β ± 1.96 × SE.

Two models were developed:

Model 1: Dependent variable—SPPB score independent variables—group, age, BMI, body weight, body height.

Model 2: Dependent variable—6MWT distance independent variables—group, age, BMI, body weight, body height.

Model fit was evaluated using R^2^, adjusted R^2^, F statistics, and standardized beta coefficients. Additionally, effect sizes (Cohen’s d) were calculated to assess the clinical relevance of between-group differences. A retrospective post hoc power analysis was performed. Given the very large effect sizes observed (Cohen’s d > 1.5), the statistical power for the primary outcomes (SPPB and 6MWT) exceeded 0.95, confirming that the sample size was adequate to detect significant between-group differences. Effect sizes (Cohen’s d) were calculated according to established thresholds.

All analyses were performed in Statistica in accordance with established statistical standards.

## 3. Results

The general characteristics of somatic features in the two examined groups indicate that there were no significant differences in BMI. None of the participants presented signs of underweight or obesity (Table 3).

Men with cardiovascular disease demonstrated markedly lower mean values for both the 6MWT distance and SPPB scores (Table 2).

Student’s *t* test for independent samples showed no significant differences between groups in age, body weight, body height, or BMI. In contrast, mean values for both 6MWT distance and SPPB score were significantly higher in men without cardiovascular disease (*p* < 0.001), confirming a very high level of statistical significance (Table 4).

The effect size (Cohen’s d) for between-group differences was very large for both SPPB (d = 1.51) and 6MWT distance (d = 1.64), indicating strong clinical relevance.

### Regression Analysis

In the regression model predicting SPPB scores (Model 1), group allocation remained a strong and independent predictor of functional capacity after adjusting for age and somatic parameters (β = −3.33; *p* < 0.001). Age was also a significant negative predictor (*p* = 0.006), whereas BMI, body weight, and body height were not significant. The model explained 47.5% of the variance (adjusted R^2^ = 0.437).

In the model predicting 6MWT distance (Model 2), group allocation was again the strongest independent predictor (β = −137.9 m; *p* < 0.001). Age also significantly reduced test performance (*p* = 0.026). BMI, body weight, and body height were not statistically significant. The model explained 46.4% of the variance (adjusted R^2^ = 0.425).

Across both study groups, the distances covered in the 6-Minute Walk Test were significantly lower than the established nationwide norms for older adults in Poland (Table 5).

All regressions are presented together with their 95% confidence intervals (Table 6 and Table 7).

## 4. Discussion

Numerous studies indicate that the ageing process contributes to a decline in functional capacity and aerobic performance [1,16]. Reduced exercise tolerance is primarily associated with a progressive decrease in maximal oxygen uptake, estimated at approximately 0.5–1.0% per year [16]. The comparison of SPPB and 6MWT outcomes in the present study revealed significant differences between the analyzed groups. Men with coronary heart disease and arterial hypertension achieved substantially lower scores in both tests compared with men without cardiovascular disease, confirming reduced functional capacity and physical performance. The very large effect size (Cohen’s d > 1.5) further demonstrates that these differences are clinically meaningful and cannot be attributed solely to demographic variation.

Regression analysis showed that lower functional capacity and exercise tolerance among residents with cardiovascular disease were independent of age, BMI, body weight, and body height, underscoring the substantial impact of cardiovascular burden on physical functioning. These findings are consistent with previous reports demonstrating that individuals with coronary heart disease exhibit poorer exercise tolerance and reduced functional performance [17]. Numerous studies also indicate that regular physical activity and structured exercise training may improve coronary microvascular dysfunction (CMD), thereby enhancing physical performance and cardiovascular function [18].

In the HULK study, which included patients over 70 years of age hospitalized for acute coronary syndromes, a six-month structured exercise program resulted in improvements in SPPB scores and gait speed [19,20]. Similar outcomes were reported by other authors, who observed enhanced functional capacity and exercise tolerance in individuals aged 70–89 years following the implementation of regular physical activity [21]. In the HYCARET project, a 12-week training program significantly increased 6MWT distance in patients with coronary heart disease aged approximately 66 years [22].

Comparable findings were reported in a study involving individuals aged 61.6 ± 10.2 years with coronary artery disease, where a five-week physical activation program significantly increased 6MWT distance (*p* < 0.001). Improvements were also observed in lipid profiles (total cholesterol, LDL, HDL, triglycerides) and resting blood pressure, confirming the beneficial role of physical activity in the secondary prevention of cardiovascular disease [23,24,25,26]. Participation in physical activity programs after myocardial infarction has also been shown to improve survival [27].

Researchers emphasize that SPPB score is strongly associated with long-term mortality, and a value ≤ 9 points is considered an indicator of reduced physical performance [28]. Despite this, functional capacity is not routinely assessed in Polish residential care homes, and physical activation programs are not systematically implemented [29]. This is reflected in the present study, where both groups of men achieved relatively low SPPB scores, with significantly poorer results in the cardiovascular disease group.

Frailty, defined as reduced physiological reserve and increased vulnerability to stressors, leads to accelerated functional decline, higher morbidity, and increased mortality. It has also become a key issue in cardiovascular medicine [30,31]. An SPPB score ≤ 5 points is considered a diagnostic threshold for frailty [13]. In the present study, the group without cardiovascular disease achieved higher scores, whereas the group with CHD and hypertension approached borderline values, suggesting an elevated risk of frailty. Previous studies reported annual mortality rates of 62% for an SPPB score of 1 point, 45% for 4.5 points, 17% for 8 points, and 12% for 9 points [32].

Epidemiological analyses consistently show that slower gait speed is a significant predictor of increased cardiovascular mortality [33,34]. The 6MWT is an independent prognostic indicator, and shorter walking distance is strongly associated with higher all-cause and cardiovascular mortality [35]. Among men aged 72–83 years, covering < 338 m in the 6MWT was associated with a 1.5-fold higher risk of death compared with those achieving ≥ 414 m [36].

In the present study, the group with better exercise tolerance achieved a mean distance of 312.6 ± 89.4 m, which is substantially lower than the nationwide Polish norms (519.7 ± 123.9 m) [15]. Even lower values were observed in the group with coronary heart disease and hypertension (169.4 ± 84.4 m). These findings suggest that the level of physical activation in both groups may be insufficient. Previous studies also indicate that residents of care homes achieve poorer 6MWT results than independently living older adults [10].

The mechanisms underlying reduced functional performance in individuals with coronary heart disease are multifactorial. First, a diminished cardiac output reserve, resulting from impaired systolic and diastolic function, limits the ability to increase stroke volume and cardiac output during exertion, directly reducing exercise tolerance [37]. Second, peripheral skeletal muscle deconditioning is frequently observed in CHD, including reduced muscle mass, lower capillary density and decreased oxidative enzyme activity, all of which impair metabolic efficiency during physical activity [38]. Additionally, microvascular dysfunction characterized by impaired coronary and peripheral vasodilatory responses leads to insufficient tissue perfusion during exercise [39]. The coexistence of these mechanisms means that even moderate physical activity imposes a disproportionately high hemodynamic and metabolic burden on individuals with CHD, which is reflected in the lower SPPB scores and shorter 6MWT distances in our study. Recent evidence highlights that cardiac rehabilitation plays a crucial role in improving physical capacity, muscle strength and exercise tolerance in individuals with cardiovascular disease. As emphasized in a recent publication, comprehensive rehabilitation programs should be considered an essential component of care for patients with coronary artery disease and hypertension, particularly in population with reduced functional capacity [40]. The markedly lower SPPB and 6MWT performance observed in our participants with cardiovascular disease supports the need for implementing structured rehabilitation interventions in residential care settings, where access to such programs is often limited.

In Poland, residential care homes operate under the Regulation of the Ministry of Labour and Social Policy (2012, with amendments in 2017 and 2018), which obliges them to provide therapeutic activities, including physical activation. In practice, however, the number of therapists and physiotherapists is insufficient, and activation programs are often not implemented systematically. Additionally, many residents decline participation, present passive attitudes, and spend most of the day in a seated position [41,42].

Taken together, the findings of this study and the available literature indicate that physical activation programs in Polish residential care homes may be insufficient, infrequent, or inadequately tailored to residents’ needs. This highlights the need for effective, regular physical activity programs aimed at prolonging independence, reducing falls, and limiting the risk of premature disability and mortality, particularly among older adults with cardiovascular disease.

### Strengths and Limitations

This study has several limitations. First, no formal a priori power calculation was performed, as the sample size was determined by the availability of eligible participants within the observation design. Although this may limit the statistical robustness of the findings, the large and consistent effect sizes observed reduce the likelihood of type II error. Second, detailed information on pharmacotherapy was not collected; in particular, the use of beta-blockers, which may blunt chronotropic and exercise responses, could have influenced functional outcomes. Third, no clinical indications of disease severity (such as NYHA functional class or left ventricular ejection fraction) were available, limiting the ability to contextualize cardiovascular impairment. Fourth, data on previous participation in cardiac rehabilitation programs were not obtained, which may also have affected functional performance. Fifth, the sample consisted exclusively of men. Which restricts the generalizability of the findings to women. Finally, the cardiovascular disease group was relatively small (n = 30), which may affect the precision of estimates.

Another important limitation is the absence of objective cardiopulmonary fitness assessment using cardiopulmonary exercise testing (CPET). Although the 6MWT is a widely used and practical submaximal test, it provides only an indirect estimate of aerobic capacity and its correlation with VO_2_ peak in older adults with cardiovascular disease is limited. CPET allows for the evaluation of key parameters such as VO_2_ peak, oxygen pulse and the VE/VCO_2_ slope, which have strong diagnostic and prognostic value in cardiovascular populations. The lack of CPET measurements should therefore be considered a significant methodological limitation [43].

Furthermore, detailed clinical characteristics such as cardiovascular risk factors, blood pressure control, severity of coronary artery disease, history of myocardial infarction revascularization, heart failure, atrial fibrillation, diabetes, renal function, neurological or musculoskeletal disorders, frailty status, medication use and physical activity levels were not collected. These data were unavailable due to organizational constraints in residential care homes and the observational nature of study. Although this approach ensured participant safety and allowed for clear group classification, it limits clinical depth of the analysis and reduces the generalizability of the findings. Furthermore, due to the cross-sectional design, the study cannot establish causal relationships. The observed differences in SPPB and 6MWT performance may be influenced by unmeasured factors such as frailty, sarcopenia, multimorbidity, depression, malnutrition, pain, institutionalization or low baseline physical activity. These variables were not available in the institutional medical records and could not be included in the analysis, which further limits the interpretability and generalizability of the findings.

Additionally, the strict inclusion and exclusion criteria applied in this study including the exclusion of residents with obesity, cognitive or physical impairments, cancer, recent myocardial infarction, infections or other contraindications may have introduced selection bias. These criteria were necessary to ensure participant safety and to obtain a relatively homogeneous sample; however, they limit the generalizability of the findings to the broader population of residential care home residents.

Despite these limitations, the study has several strengths. A major strength of this study is the use of reliable, validated tools for assessing functional capacity and physical performance, as well as the inclusion of a homogeneous group of older men. Recruiting such a group is particularly challenging, as older men participate in research less frequently than women, which strengthens the value of the present findings. The use of both multivariable regression analysis and effect size estimation (Cohens’s d) further supports the robustness and clinical relevance of the results.

The findings highlight substantial gaps in preventive care and physical activation among residents of residential care homes. These results have important clinical implications for designing and implementing effective physical activity-based health promotion programs aimed at supporting successful ageing, maintaining independence and reducing healthcare and hospitalization costs. It should be noted that the cross-sectional design does not allow causal inferences, and future longitudinal research is needed to assess trajectories of functional changes and their relationship with physical activity.

## 5. Conclusions

1. Residential care home residents with arterial hypertension and coronary heart disease demonstrate significantly lower functional capacity and exercise tolerance compared with individuals without cardiovascular disease.
2. Both study groups achieved results markedly below nationwide reference values, which is likely associated with insufficient implementation of physical activation programs in Polish residential care homes.
3. Targeted physical activity programs should be introduced in residential care homes, with particular emphasis on individuals with cardiovascular disease, to improve functional capacity, physical performance, and overall health outcomes.

### Implications for Practice

Implementing structured physical activity programs in residential care settings is essential for improving functional capacity and exercise tolerance among older adults, particularly those with cardiovascular disease. Regular assessment of physical performance using tools such as SPPB and 6MWT should become a routine component of clinical monitoring in DPS facilities, enabling early identification of functional decline and frailty risk. Introducing individualized, progressive exercise interventions—supervised by qualified physiotherapists—may help slow functional deterioration, enhance mobility, and reduce cardiovascular risk. Strengthening staffing levels and ensuring consistent access to therapeutic services are critical for achieving these goals. Integrating evidence-based activation programs into daily routines could support healthy aging, prolong independence, and reduce healthcare utilization among older residents.

## Figures and Tables

**Table 1:** BMI categories.

Category	BMI
Underweight	<18.5 kg/m^2^
Normal weight	18.5–24.9 kg/m^2^
Overweight	25–29.9 kg/m^2^
Obesity	>30 kg/m^2^

BMI categories were defined according to WHO criteria [14].

**Table 2:** Shapiro–Wilk test results—assessment of distribution normality in both groups.

Variable	Group I (*p*-Value)	Group II (*p*-Value)	Distribution
Age [years]	0.000	0.000	skewed
Weight [kg]	0.871	0.385	normal
Body height [cm]	0.581	0.158	normal
BMI [kg/m^2^]	0.061	0.077	normal
SPPB points	0.061	0.280	normal
6MWT [m]	0.368	0.230	normal

SPPB—Short Physical Performance Battery; 6MWT—6-Minute Walk Test; Group I—44 men without diagnosed cardiovascular disease; Group II—30 men with diagnosed and treated CHD and arterial hypertension.

**Table 3:** The general statistical characteristics.

Variable	Group I M ± SD	Group II M ± SD
Age [years]	70.8 ± 6.1	71.8 ± 6.7
Weight [kg]	69.2 ± 10.1	70.6 ± 8.2
Body height [cm]	166.8 ± 9.3	168.2 ± 9.3
BMI [kg/m^2^]	24.8 ± 2.8	25.2 ± 2.9
SPPB points	8.7 ± 2.1	5.2 ± 2.5
6MWT [m]	312.6 ± 89.4	168.4 ± 84.4

M—mean; SD—standard deviation; Group I—44 men without diagnosed cardiovascular disease; Group II—30 men with diagnosed and treated CHD and arterial hypertension.

**Table 4:** Analysis of the significance of differences in the mean values of the examined variables between the group of healthy men and the group of men with cardiovascular diseases.

Variables	Group	M ± SD	Me	Min–Max	*p*
Age [years]	1	70.8 ± 6.1	68	65–80	0.8
	2	71.8 ± 8.2	69.5	65–80	
Weight [kg]	1	69.2 ± 10.1	65	49–90	0.527
	2	70.6 ±8.2	69	49–90	
Body height [cm]	1	166.8 ± 9.3	167.5	149–185	0.529
	2	168.2 ± 9.3	168	155–188	
BMI [kg/m^2^]	1	24.8 ± 2.8	24.7	19.9–29.8	0.531
	2	25.2 ± 2.9	25.6	20.3–29.6	
SPPB points	1	8.7 ± 2.1	9	4–12	*p* < 0.001
	2	5.2 ± 2.5	5	1–11	
6MWT distance [m]	1	312.6 ± 89.4	321.5	153–550	*p* < 0.001
	2	169.4 ± 84.4	170	32–380	

SPPB—Short Physical Performance Battery; Group I—44 men without cardiovascular disease; Group II—30 men with CHD and arterial hypertension; M—mean; SD—standard deviation; Me—median; Min—minimum; Max—maximum.

**Table 5:** Reference to nationwide norms for the 6MWT distance in older adults.

Variables	Group	M ± SD	*p*
6MWT distance [m]	1	312.6 ± 89.4	*p* < 0.001
	norms (*N* = 1159)	519.7 ± 123.9	
6MWT distance [m]	2	169.4 ± 84.4	*p* < 0.001
	norms (*N* = 1159)	519.7 ± 123.9	

M—mean; SD—standard deviation; Group 1—44 men without cardiovascular disease; Group 2—30 men with CHD and arterial hypertension; *N*—sample size.

**Table 6:** Multiple linear regression for SPPB.

Variable	Coefficient (SE)	Lower 95%CI for β	Upper 95%CI for β	*p*-Value
Age (years)	−0.09 (0.02)	−0.13	−0.05	<0.001
BMI (kg/m^2^)	−0.03 (0.04)	−0.11	0.05	0.42
Group II	−2.10 (0.35)	−2.80	−1.40	<0.001

**Table 7:** Multiple linear regression for 6MWT.

Variable	Coefficient (SE)	Lower 95%CI for β	Upper 95%CI for β	*p*-Value
Age (years)	−4.8 (1.2)	−7.2	−2.4	<0.001
BMI (kg/m^2^)	−1.5 (2.4)	−6.3	3.3	0.53
Group II	−115 (18)	−150	−82	<0.001

## Data Availability

The data supporting the findings of this study are available from the corresponding author upon reasonable request.
